# Cell‐based and antibody‐mediated immunotherapies directed against leukemic stem cells in acute myeloid leukemia: Perspectives and open issues

**DOI:** 10.1002/sctm.20-0147

**Published:** 2020-07-13

**Authors:** Peter Valent, Karin Bauer, Irina Sadovnik, Dubravka Smiljkovic, Daniel Ivanov, Harald Herrmann, Yüksel Filik, Gregor Eisenwort, Wolfgang R. Sperr, Werner Rabitsch

**Affiliations:** ^1^ Department of Internal Medicine I, Division of Hematology and Hemostaseology Medical University of Vienna Vienna Austria; ^2^ Ludwig Boltzmann Institute for Hematology & Oncology Medical University of Vienna Vienna Austria; ^3^ Department of Radiation Oncology Medical University of Vienna Vienna Austria; ^4^ Department of Internal Medicine I, Stem Cell Transplantation Unit Medical University of Vienna Vienna Austria

**Keywords:** bi‐specific antibodies, CAR‐T and CAR‐NK cell‐therapy, immune‐checkpoints, leukemic stem cells, precision medicine, stem cell resistance

## Abstract

Despite new insights in molecular features of leukemic cells and the availability of novel treatment approaches and drugs, acute myeloid leukemia (AML) remains a major clinical challenge. In fact, many patients with AML relapse after standard therapy and eventually die from progressive disease. The basic concept of leukemic stem cells (LSC) has been coined with the goal to decipher clonal architectures in various leukemia‐models and to develop curative drug therapies by eliminating LSC. Indeed, during the past few years, various immunotherapies have been tested in AML, and several of these therapies follow the strategy to eliminate relevant leukemic subclones by introducing LSC‐targeting antibodies or LSC‐targeting immune cells. These therapies include, among others, new generations of LSC‐eliminating antibody‐constructs, checkpoint‐targeting antibodies, bi‐specific antibodies, and CAR‐T or CAR‐NK cell‐based strategies. However, responses are often limited and/or transient which may be due to LSC resistance. Indeed, AML LSC exhibit multiple forms of resistance against various drugs and immunotherapies. An additional problems are treatment‐induced myelotoxicity and other side effects. The current article provides a short overview of immunological targets expressed on LSC in AML. Moreover, cell‐based therapies and immunotherapies tested in AML are discussed. Finally, the article provides an overview about LSC resistance and strategies to overcome resistance.

## Graphical abstract

Expression of cell surface target antigens on leukemic stem cells (LSC) in acute myeloid leukemia (AML) (left image) and on normal hematopoietic stem cells (right panel). Most of the clinically relevant surface target antigens are not only expressed on AML LSC but also in normal stem cells. In several instances, expression levels are higher on AML LSC than on normal stem cells providing a (small) therapeutic window. Only a few potential surface targets, such as CLL1 (CD371), are selectively (aberrantly) expressed on AML LSC but not on normal stem cells.

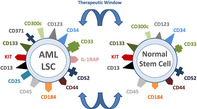

**Significance statement**

Antibody‐based and cell‐based immunotherapies are emerging therapeutic approaches in applied hematology. However, although several attempts were made and several molecular markers have been identified, no curative immunotherapy is available in acute myeloid leukemia (AML). One explanation is that leukemic stem cells (LSC) are often resistant and are residing in several different fractions of the AML‐clone. This article discusses novel markers and targets expressed on LSC in AML, with emphasis on the potential value of these antigens in the context of antibody‐based and/or cell‐based therapies. Moreover, the article discusses various limitations and possible pitfalls of such therapies.

## 1 INTRODUCTION

Acute myeloid leukemia (AML) is a life‐threatening malignancy characterized by an uncontrolled expansion of myeloid blast cells in the bone marrow (BM) and peripheral blood. Clinical presentation and courses in AML vary among patients, depending on the type of AML, molecular and immunological features of clonal cells, patient‐related variables such as age or comorbidities, and response to treatment.^1^, ^2^, ^3^, ^4^, ^5^, ^6^, ^7^, ^8^ In most patients with *de novo* AML, induction poly‐chemotherapy produces complete remission (CR), and many of these CR‐patients can be kept in continuous CR by introducing consolidation therapy. Notably, consolidation therapy is applied with the aim to eliminate most or all of the remaining leukemic (stem) cells after remission‐induction therapy.^1^, ^7^ The most effective consolidation‐therapy is allogeneic hematopoietic stem cell transplantation (HSCT).^1^, ^7^ This form of consolidation is typically offered to high‐risk AML patients and those who relapse. However, not all patients are fit enough for HSCT, and elderly patients may not even tolerate multiple cycles of intensive chemotherapy. In patients who fail to respond to initial induction chemotherapy or relapse after therapy, the prognosis is grave.

In the past 20 years, treatment of several AML variants has substantially improved which is mainly due to the availability of new (targeted) drugs, improved diagnostics, better selection of patients for various therapies (personalized medicine), and advances in HSCT strategies.^3^, ^4^, ^5^, ^6^, ^7^, ^8^ Still, however, many patients relapse or have resistant disease. As a result, AML research is still moving and seeking new ways to improve interventional therapies.

One strategy is to involve the immune system and to use targeted drugs and/or specific lymphoid killer cells that can attack and eliminate all AML cells (all subclones). Another related strategy is to kill all AML cells that have a particular ability to propagate the malignancy for longer or even unlimited time‐periods.

The concept of leukemic stem cells (LSC) has been established to explain subclonal architectures and hierarchies in acute and chronic leukemias.^9^, ^10^, ^11^, ^12^, ^13^, ^14^ In fact, leukemias are considered to be organized in cellular hierarchies that include (a) more mature stages of leukemic cells that have only a limited ability to divide and then disappear and (b) LSC that have an unlimited capacity to divide and to self‐renew, thereby propagating the malignancy for unlimited time‐periods.^9^, ^10^, ^11^, ^12^, ^13^, ^14^ The LSC concept predicts that any type of targeted therapy or other treatment approach can only be curative when (a) the approach does not only attack the bulk of AML cells but also LSC, (b) all LSC in all subclones of the disease exhibit the target(s), and (c) treatment is independent of or overcomes LSC resistance.^14^, ^15^, ^16^, ^17^, ^18^, ^19^ Initial studies suggested that AML LSC reside primarily in a phenotypically more immature, CD34^+^/CD38^−^/Lin^−^ subset of the AML clones.^9^, ^10^, ^11^ However, depending on molecular features, aberration profiles, and the diagnostic subvariant of AML, LSC are also detectable in a (phenotypically) more mature CD34^+^/CD38^+^ subset or even in CD34‐negative AML cell subsets.^20^, ^21^

In the past few years, the LSC concept has attracted more and more attention, and a rapidly expanding number of studies have discovered and validated distinct molecular targets and target‐pathways in these cells.^9^, ^10^, ^11^, ^12^, ^13^, ^14^, ^15^, ^16^, ^17^, ^18^, ^19^ With regard to specific cell therapies and immunotherapies, surface membrane targets are of great interest. In fact, such molecules are increasingly used to establish AML‐eradicating approaches. However, thus far, only a very few cell membrane antigens expressed specifically on LSC, but not on normal lymphohematopoietic stem cells, have been identified.

In this article, target expression profiles of AML LSC are reviewed, and possibilities to design specific LSC‐eradicating therapies in AML are discussed, with special focus on antibody‐based and cell‐based therapy. In addition, the article reviews the multiple forms and mechanisms of LSC resistance and ways to overcome resistance.

## 2 CELL SURFACE MEMBRANE PHENOTYPE OF AML LSC

The three classic “stem cell features” of AML LSC are (a) their archaic self‐renewal capacity, (b) their long‐term disease‐propagating capability *in vivo*, and (c) their capacity to survive well in extreme situations, toxic exposures, and total quiescence.^15^, ^16^ To document the first two stem cell features, (a) + (b), it is standard to inject LSC into immunocompromised mice and to measure long‐term engraftment. In initial xenotransplant experiments, severe combined immunodeficient (SCID) mice or nonobese SCID (NOD/SCID) mice were used.^9^, ^10^, ^11^ In these studies, leukemic engraftment was only produced by CD34^+^/CD38^−^ AML cells but not by CD34^+^/CD38^+^ cells.^9^, ^10^, ^11^ Based on these data, AML LSC were believed to reside selectively in a CD34^+^/CD38^−^ fraction of AML cells. Later, it was found that the residual immune system of NOD/SCID mice can destroy CD38^+^ AML cells and that after blocking the residual immune system, also CD38^+^ AML LSC fractions can produce leukemic engraftment in the BM of NOD/SCID mice.^20^ As a consequence, highly immunodeficient (and thus more permissive) mouse subsets were used and were soon accepted as a new standard to study AML LSC. One such standard‐strain is NSG, a NOD/SCID mouse model lacking a functional interleukin‐2 receptor gamma‐chain. In most AML subsets, NSG‐engrafting AML LSC are detectable in both, the CD34^+^/CD38^−^ and CD34^+^/CD38^+^ subsets of the malignant clone.^20^ In the blast phase (blast crisis) of chronic myeloid leukemia and in acute lymphoblastic leukemia, NSG‐engrafting stem cells are also found in both, the CD34^+^/CD38^−^ and CD34^+^/CD38^+^ subfraction of leukemic cells.^22^, ^23^ By contrast, normal hematopoietic stem cells are CD34^+^/CD38^−^ cells. The fact that AML LSC reside in multiple fractions of CD34^+^ cells, and sometimes (rarely) even in CD34‐negative cell populations in some AML variants,^21^ is an important issue when considering effective cell therapies and immunotherapies. Another important point is the molecular complexity and the related subclone formation in AML.^15^, ^16^, ^17^, ^18^, ^19^

So far, little is known about phenotypic properties of AML LSC. Based on LSC features described above, a surface marker or target can only be considered as being “expressed on AML LSC,” when it is detected on most or all CD34^+^/CD38^−^ and most or all CD34^+^/CD38^+^ cells. Cell surface membrane structures that fulfill these criteria in most major AML variants and in most (but not all) patients are Siglec‐3 (CD33), Hermes (CD44), CD96, KIT (CD117), the interleukin‐3 receptor alpha‐chain (CD123), AC133 (CD133), FLT3 (CD135), and CXCR4 (CD184) (Table 1).^24^, ^25^, ^26^, ^27^, ^28^, ^29^, ^30^, ^31^, ^32^, ^33^ In approximately 50% of all patients with de novo AML, both the CD34^+^/CD38^+^ and the CD34^+^/CD38^−^ cell subsets express the C‐type lectin protein‐1, CLL‐1 (CD371).^34^ By contrast, normal hematopoietic (CD34^+^/CD38^−^) stem cells do not express CLL‐1.^34^ Normal hematopoietic stem cells also lack or express only low amounts of CD25, CD26, CD33, and IL‐1RAP.^35^, ^36^, ^37^

**TABLE 1:** Expression of cell surface targets on CD34^+^/CD38^−^ and CD34^+^/CD38^+^ stem and progenitor cells in AML and comparison to normal hematopoietic stem cells (HSCs)

Antigen^a^	CD	Expression on stem/progenitor cells^b^			Therapeutic concepts tested / developed in preclinical studies or in clinical trials in AML
		HSC	AML LSC and progenitor cells		
		CD34^+^/CD38^−^	CD34^+^/CD38^−^	CD34^+^/CD38^+^	
IL‐2RA	CD25	−	+/−	+/−	Toxin‐ligand
Siglec‐3	CD33	+	+	+	AbC, BiTE/TriKE, DART, CAR
Hermes	CD44	+	+	+	Ab, CAR
IAP	CD47	+	+	+	Ab, CAR^d^
Campath1	CD52	+/−	+/−	+/−	Ab
KIT	CD117	+	+	+	Ab, TKI, CAR
IL‐3RA	CD123	+	+	+	Ab, Ligand‐C, BiTE, DART, CAR
PROM1	CD133	+	+	+	Ab, AbC, CAR
FLT3	CD135	+/−	+	+	TKI, BiTE, CAR
CXCR4	CD184	+	+	+	Ab
PD‐L1	CD274	+/−	+/−^c^	+/−^c^	Ab
CLL‐1	CD371	−	+/−	+	Ab, CAR
IL‐1RAP	n.c.	−	+/−	+	Ab

^a^ Data refer to the available literature and/or data obtained by multicolor flow cytometry in the labs of the authors.

^b^ Score: +, strongly expressed on most or all cells; +/− weak expression on most cells or expressed on subsets of cells (10%‐50%); −/+, weak expression on cells or expressed on cells in a small subset of donors; −, not expressed on stem cells.

^c^ PD‐L1 expression can be induced or enhanced by exposure of AML cells to IFN‐G and/or TNF‐A.

^d^ So far developed mainly in solid tumors.

Abbreviations: Ab, (blocking or attacking) antibody; AbC, antibody‐toxin/cytostatic‐conjugate; AML, acute myeloid leukemia; BiTE, bi‐specific T cell engager; CAR, chimeric antigen receptor; CLL‐1, C‐type lectin‐like molecule‐1; CXCR4, C‐X‐C chemokine receptor type 4; DART, dual affinity retargeting agent; HSCs, hematopoietic stem cells; IAP, integrin associated protein; IFN, interferon; IL‐1RAP, interleukin‐1 receptor accessory protein; IL‐2RA, interleukin‐2 receptor alpha chain; IL‐3RA, interleukin‐3 receptor alpha chain; Ligand‐C, ligand‐toxin‐conjugate; n.c., not yet clustered; PD‐L1, programmed cell death‐ligand 1; PROM1, prominin‐1; TKI, tyrosine kinase inhibitor; TNF, tumor necrosis factor; TriKE, tri‐specific killer engager.

Additional surface membrane antigens expressed on CD34^+^ or CD34^+^/CD38^−^ stem cells in AML (in subsets of patients) include, among others, CD93 and CD96.^30^, ^38^, ^39^ In a smaller subset of patients, AML LSC exhibit CD25, CD26, and/or IL‐1RAP.^36^, ^37^ AML stem cells also display Campath‐1 (CD52).^40^ However, this antigen is also expressed on normal hematopoietic stem cells (Table 1).

Finally, AML LSC display various immune checkpoint antigens, including CD47, PD‐L1 (CD274), and the CTLA4 ligands CD80 and CD86 (Table 2).^41^, ^42^ Whereas CD47 is abundantly and almost invariably expressed on LSC (and on normal stem cells), expression of PD‐L1 is commonly weak or lacking on AML LSC (Table 2). However, expression of PD‐L1 can be induced or augmented on AML cells (including LSC) by exposure to certain cytokines, such as interferon‐gamma (IFN‐G) and/or tumor necrosis factor‐alpha (TNF‐A). Depending on the cell type, and culture condition, both cytokines are required to induce a substantial upregulation of PD‐L1 on AML cells (Figure S1).

**TABLE 2:** Expression of checkpoint molecules on CD34^+^/CD38^−^ and CD34^+^/CD38^+^ stem and progenitor cells in AML^a^

Antigen	CD	Antigen expression on stem/progenitor cells in AML	
		CD34^+^/CD38^−^	CD34^+^/CD38^+^
T44	CD28	−	−
IAP	CD47	+++	+++
B7‐1	CD80	+/−	+/−
BL11	CD83	+/−	+/−
B7‐2	CD86	+/−	+/−
MDR‐1	CD243	−	−
PD‐L2	CD273	−	−
PD‐L1	CD274	+	+
PD‐1	CD279	−	−
TIM‐3	CD366	+/−	+/−

^a^ Data refer to results obtained by multicolor flow cytometry in the lab of the authors. Scoring system: staining index (SI) was calculated (ratio of median fluorescence intensities obtained with specific antibody and isotype‐matched control antibody) and scored according to the following grading system: −, SI < 1.30; +/−, SI 1.31‐3.00; +, SI 3.01‐10.00; ++, SI 10.01‐100; +++, SI > 100.

Abbreviations: AML, acute myeloid leukemia; IAP, integrin‐associated protein; MDR‐1, multidrug resistance protein‐1; PD‐1, programmed cell death‐1; PD‐L1, programmed cell death‐ligand 1; PD‐L2, programmed cell death‐ligand 2; TIM‐3, T cell immunoglobulin and mucin domain‐containing protein‐3.

## 3 TARGETING AML LSC WITH ANTIBODY‐BASED DRUGS

So far, only a few antibody‐based compounds have been developed and evaluated clinically for their application and efficacy in patients with AML.^43^, ^44^, ^45^ This can be explained by the fact that it is difficult to identify cell surface membrane antigens that are expressed selectively on AML LSC but not on normal hematopoietic stem cells. Indeed, most targets are expressed on normal BM stem cells as well, but expression levels are often lower compared to LSC, providing (hope for) a clinically sufficient or even robust therapeutic window.^25^, ^29^, ^45^, ^46^, ^47^ Such antigens include, among others, CD25, CD26, CD33, CD93, CD96, CD123, IL‐1RAP, and CLL‐1. However, of all these targets, only a few have been developed clinically. Siglec‐3 (CD33) was identified quite early as a potentially valuable surface target that is expressed on AML cells and on AML LSC in excess over normal stem cells.^25^, ^29^, ^45^

Gemtuzumab ozogamicin (GO) is a fully humanized CD33 antibody that is conjugated to the toxic drug calicheamicin through a chemical linker.^43^, ^44^, ^45^ Initial studies have shown that GO can induce remission in chemotherapy‐refractory or relapsed AML and in the year 2000, the drug was approved by the FDA. However, in subsequent clinical trials, the benefit of GO in relapsed/refractory AML could not be confirmed.^46^ In addition, GO was found to produce long‐lasting cytopenias as well as veno‐occlusive liver disease. More recent studies have demonstrated that therapy with (less toxic doses of) GO in combination with chemotherapy can indeed improve the overall survival (OS) in AML patients with favorable cytogenetics but is less efficacious in those with poor cytogenetic features.^47^, ^48^, ^49^ Moreover, GO has recently been reported to improve the outcome in elderly patients with AML.^50^ The clinical benefit was especially demonstrable in AML patients in whom blast cells (CD34^+^ cells) exhibited CD33.^51^ Although GO was transiently removed from the market (since 2010), the FDA has (re)approved GO for treatment of newly diagnosed AML as adjunct to standard chemotherapy in 2017.^52^ Whereas a certain therapeutic window is demonstrable with GO, the dose applied is important, especially when combined with induction poly‐chemotherapy. Notably, lower doses of GO can in part reduce myelotoxicity (prolonged cytopenia).

In recent years, substantial efforts have been made to develop more potent CD33‐targeting antibody‐based drugs. Vadastuximab talirine (SGN‐CD33A) is a drug‐conjugate that contains a humanized anti‐CD33 antibody and a DNA‐targeting cytotoxic compound.^53^, ^54^ SGN‐CD33A has been described to be more potent in killing AML blasts in chemotherapy‐resistant disease compared to GO.^54^, ^55^, ^56^ However, the therapeutic window is relatively small, and when combined with chemotherapy, hematologic toxicity is substantial.^54^, ^55^, ^56^ The phase 3 study of SGN‐CD33A was prematurely closed because of toxicity in 2017.

In the recent past, several attempts have been made to develop drugs attacking AML LSC through the IL‐3RA (CD123). An interesting approach is to conjugate the ligand, IL‐3, with a cytotoxic drug. The diphtheria toxin DT_388_/IL‐3 fusion‐protein is such an agent.^57^, ^58^, ^59^ A derivative of this compound, DT_388_ IL‐3[K116W], was developed by conjugating the toxin to a modified IL‐3 protein, resulting in increased binding‐affinity.^60^ Subsequent studies have shown that DT_388_ IL‐3[K116W] is more effective than DT_388_ IL‐3 in killing AML blasts.^60^ Whether this difference is relevant *in vivo* in patients with AML remains uncertain. Efficacy of these agents presumably depends on expression of the IL‐3R on AML LSC. For both agents, however, the therapeutic window is small as normal stem cells also display (low amounts of) CD123. Another IL‐3‐toxin fusion protein is SL‐401 (tagraxofusp). In initial trials, this drug produced clinically meaningful responses in a smaller subset of patients with heavily pretreated AML.^61^, ^62^ However, the clinical value of tagraxofusp in AML remains uncertain. On the other hand, tagraxofusp has recently been reported to be a most effective targeted agent in the treatment of patients with plasmacytoid dendritic cell neoplasms.^63^ The drug received an approval for this new indication by the FDA in 2018.

Another approach to target the alpha chain of the human IL‐3R (CD123) is to develop antibodies that can bind to CD123 with high affinity and can thereby inhibit the binding and the effects of IL‐3 on AML cells.^64^, ^65^ The CD123 antibody 7G3 identifies the N‐terminal domains of CD123, and acts as potent IL‐3R‐antagonist.^64^ This antibody, 7G3, can produce substantial growth‐inhibitory effects *in vitro* and *in vivo* on AML cells and AML LSC, while exerting less toxic effects on normal hematopoietic stem cells.^64^ In subsequent studies, 7G3 was humanized and further engineered for optimal antibody‐dependent cytotoxicity, which resulted in the development of the targeted antibodies CSL360 and CSL362.^65^, ^66^ Both agents were found to be effective in blocking the growth of AML blasts. However, when evaluating efficacy of CSL360 in a clinical phase I trial in chemotherapy‐refractory AML, no major antileukemic activity was observed in most patients enrolled.^66^ In a recent trial, talacotuzumab, a humanized anti‐CD123 antibody, was administered in combination with decitabine in older AML patients not eligible for poly‐chemotherapy.^67^ However, the drug combination did not result in a better outcome compared to decitabine alone.^67^

Other surface targets that have been considered in the context of AML and antibody‐mediated elimination of LSC include CD44, CD45, CD47, CD93, CD96, CD157, CD330f, and CLL‐1.^8^, ^30^, ^31^, ^32^, ^33^, ^34^, ^38^, ^43^, ^68^, ^69^, ^70^ However, so far, no major AML‐eradicating activity was obtained in clinical trials.

There are also a number of radiolabeled antibodies that have been considered for application in AML. Among these agents are ^131^I‐, ^213^Bi‐, or ^225^Ac‐conjugated anti‐CD33 or CD45 antibodies, and ^188^Rhe‐labeled CD66 antibody.^71^, ^72^, ^73^, ^74^ Unfortunately, these antibodies usually produce substantial hematologic toxicities, including prolonged cytopenia (long‐term aplasia) which is due to the accumulation of these agents in various hematopoietic tissues thereby affecting also normal hematopoietic stem and progenitor cells by cross‐radiation. As a result, such antibodies have to be applied in conjunction with a subsequent HSCT.^71^, ^72^, ^73^, ^74^ However, so far, it remains unknown whether conditioning regimens with radiolabeled cytotoxic antibodies can eliminate more LSC and can thus improve post‐HSCT outcomes in patients with chemotherapy‐refractory AML.

## 4 TARGETING AML LSC BY AGENTS DIRECTED AGAINST IMMUNE CHECKPOINT TARGETS

Several immune checkpoint molecules, such as CD28, CD47, PD‐L1, PD‐L2, or TIM3, have been implicated in the resistance of malignant/leukemic (stem) cells in diverse hematopoietic malignancies. Moreover, it has been described that AML cells and AML LSC can express one or more of these immune checkpoint antigens (Table 2, Figure S1).^31^, ^41^, ^42^ As a result, several treatment concepts have been proposed with the aim to overcome immunological resistance by blocking these checkpoint molecules in AML.^75^, ^76^, ^77^, ^78^ However, responses to these antibody‐based therapies appear to be variable and are often transient. It has also been described that certain anti‐leukemic drugs, such as the hypomethylating agents, can promote PD‐L1 expression in neoplastic cells.^79^, ^80^ Therefore, antibodies targeting PD‐L1 have been combined with these therapeutics in clinical trials in patients with myelodysplastic syndrome (MDS) and AML.^81^

Overall, little is known about mechanisms contributing to the expression of PD‐L1 in AML LSC. Three principle mechanisms may be responsible: first, cytokines, especially IFN‐G and TNF‐A, are considered to induce/promote expression of this checkpoint antigen (Figure S1).^41^, ^78^, ^79^, ^82^ A second mechanism is oncoprotein‐dependent expression of PD‐L1: in particular, various oncoproteins and related signaling cascades, such as the JAK‐STAT pathway or the MYC‐pathway, can contribute to PD‐L1 expression on malignant (stem) cells.^41^, ^83^ Finally, as mentioned, some of the anti‐AML drugs applied, like the hypomethylating agents, can promote expression of PD‐L1 on AML cells.^79^, ^80^ Subsequently, demethylating drugs, such as azacytidine, were combined with PD‐L1‐targeting antibodies, with the hope to increase antileukemic effects. However, a trial combining azacytidine with nivolumab showed only modest results.^81^

In the recent past, molecular mechanisms contributing to cytokine‐induced expression of PD‐L1 on AML (stem) cells have been examined. In these investigations, the BRD4‐MYC axis and the JAK‐STAT pathway were described as major drivers of PD‐L1 expression.^41^, ^82^, ^83^ Correspondingly, the BRD4/MYC‐targeting compound JQ1 was found to block IFN‐G‐induced expression of PD‐L1 on LSC.^41^, ^82^ However, it remains unknown whether targeting of PD‐L1 expression on AML LSC by BRD4/MYC inhibitors is relevant in clinical contexts. It is noteworthy here that these inhibitors also exert strong direct antineoplastic effects on AML LSC.^84^, ^85^

Other interesting checkpoint molecules in the AML context are CD47, the CTLA4 ligands CD80 and CD86 and TIM‐3.^31^, ^82^, ^86^ In almost all patients with AML, LSC express CD47, and in many cases these cells also display CD80, CD86, and TIM3 (Figure S2). CD47 is of special interest, because it is a “do not eat me antigen” that mediates the escape of AML (stem) cells from phagocytosis by macrophages.^31^, ^87^ As a result, blocking of CD47 on LSC enhances their uptake and elimination by macropahages.^31^, ^87^ First clinical data with CD47 antibody Hu5F9‐G4 (Phase 1b trial) are also available and suggest that such therapy is well tolerated and is effective alone or when combined with azacytidine in AML patients.^88^

Another interesting checkpoint is TIM‐3. This antigen and its ligand, Galectin‐9, act in a constitute autocrine loop that appears to be essential for LSC survival in AML.^86^ The clinical implication of this observation remains unknown. Overall, the clinical perspectives of checkpoint inhibitors in AML remain unclear. Whereas combination strategies may be promising, the real problem may be LSC resistance in AML.

## 5 TARGETING OF AML LSC BY APPLYING BI‐ OR TRI‐SPECIFIC ANTIBODIES

The so‐called bi‐specific antibodies are engineered compounds that consist of antigen recognition sites from two or more antibodies, thus providing binding‐interactions with two or multiple target antigens. These antibodies have certain advantages compared to conventional antibodies or monospecific toxin‐conjugates. For example, several of these bi‐specific antibody‐type drugs are able to recruit lymphoid killer cells onto the malignant target cells.^82^, ^89^, ^90^ Bi‐specific antibodies can also be directed against key checkpoint molecules, such as PD‐L1, PD‐1, or CD47. A clinically important point is that in most instances, bi‐specific antibodies do not need to internalize for therapeutic efficacy as their major task is to recruit immune cells for killing LSC.^89^, ^90^

There are several types of bi‐specific antibodies applied in clinical hematology.^82^, ^89^, ^90^, ^91^, ^92^, ^93^ A detailed discussion of these compounds is beyond the scope of this article. Classic bi‐specific T cell‐engagers (BiTEs) are recombinant fusion‐antibodies constructed to contain two single‐chain variable fragments arranged on a polypeptide‐linker. By contrast, dual affinity retargeting (DART) antibodies are diabodies consisting of heavy‐ and light‐chain variable domains of two antigen‐specificities linked to two polypeptide chains. The first successful bi‐specific antibody applied broadly in clinical hematology was blinatumomab, a BiTE that binds CD3 and CD19 and thereby promotes T cell‐induced killing of (normal and neoplastic) B lymphocytes.^91^

In the past few years, several efforts have been made to develop effective bi‐specific antibodies for the treatment of AML.^92^, ^93^, ^94^, ^95^, ^96^, ^97^, ^98^, ^99^, ^100^, ^101^, ^102^ Such antibodies are usually designed to recruit T cells and to recognizing one of the following surface target antigens on AML (stem) cells: CD33, CD123, and CD371 (CLL‐1).^91^, ^92^, ^93^, ^94^, ^95^, ^96^, ^97^, ^98^, ^99^, ^100^, ^101^

Currently, the efficacy of these agents is evaluated in clinical trials: CD33‐directed bi‐specific antibodies examined in the AML context, are, among others, the CD33/CD3 antibody constructs AMG330 and AMG673, the tetravalent CD33/CD3 tandem‐diabody AMV564, and the humanized single‐chain bi‐specific CD33/CD3 construct GEM333.^48^, ^82^, ^103^, ^104^, ^105^

Bi‐specific antibodies directed against CD123 on AML LSC include, among others, the CD123/CD3 DART flotetuzumab, the CD123/CD3 bi‐specific IgG1 antibody JNJ‐63709178, and the CD123‐targeting bi‐specific antibody XmAb14045.^82^ Flotetuzumab was found to induce measurable antileukemic effects in 8/14 patients with treatment‐refractory AML, and in two of these cases a CR was obtained.^106^ The human IgG1 bi‐specific antibody‐construct MCLA‐117 recognizing CLL‐1 is also currently tested in AML patients in clinical studies. So far, however, little is known about clinical efficacy and toxicities of these BiTEs, DARTs, and other immune‐cell‐recruiting agents in AML.^48^, ^82^ One recurrent problem seems to be a cytokine release syndrome (CRS). Additional side effects are, among others, cytopenia and liver toxicity. However, in general, the BiTEs and DARTs currently tested in the AML context, appear to be rather well tolerated with acceptable adverse event profiles, although long‐term safety data are not yet available.

## 6 TARGETING AML LSC WITH CHIMERIC ANTIGEN RECEPTOR BASED THERAPEUTICS

During the past 5 years, several brave attempts have been made to establish anti‐AML therapies by infusing immune cells expressing genetically adapted chimeric antigen receptors (CARs) (CAR‐T or CAR‐NK).^107^, ^108^, ^109^, ^110^, ^111^, ^112^, ^113^, ^114^, ^115^ In general, CARs can be constructed against various leukemia‐related molecules for which an antibody is available, and the introduced CAR‐based modification of T or NK cells can redirect these cells against leukemic (stem) cells in a HLA‐independent manner. Several different cell surface membrane antigens, displayed by AML LSC, may serve as potential CAR‐T or NK‐cell targets. These antigens are (among others) CD33, CD44, CD123, CD135 (FLT3), CD371 (CLL‐1), and Lewis Y (LeY).^48^, ^82^, ^107^, ^108^, ^109^, ^110^, ^111^, ^112^, ^113^, ^114^, ^115^, ^116^ So far, however, only a few CAR‐based approaches have been translated from preclinical studies into clinical trials in AML. In one report, a patient with a heavily pretreated (therapy‐resistant) AML was infused with anti‐CD33 CAR‐T cells in a phase I trial.^107^ After having received CAR‐T cells, this patient developed a CRS as well as prolonged cytopenia, and despite blast cell clearance, a relapse of AML was reported several weeks after the start of therapy.^107^ In 2013, Ritchie et al reported on a phase I trial using CAR‐anti‐LeY‐T cells in AML.^115^ After infusion, these CAR‐T cells were detectable for several months. In this study, the CAR‐T cells were relatively well tolerated without major toxicity, except cytopenia. However, only three patients responded, and after a couple of months (1‐23 months) all patients relapsed. Other CAR‐T cell‐based therapies are currently being prepared in AML in preclinical studies or are tested in phase I clinical trials.^82^, ^107^, ^108^, ^109^, ^110^, ^111^, ^112^, ^113^, ^114^, ^115^, ^116^, ^117^ Based on the observation that AML LSC display aberrantly expressed cell surface target antigens and the encouraging preclinical data with CAR‐T cells recognizing CD123, CLL‐1, FLT3, and other surface molecules, there is hope that more specific and more effective CAR‐T cell based approaches will be developed for AML patients in the future.^82^, ^107^, ^108^, ^109^, ^110^, ^111^, ^112^, ^113^, ^114^, ^115^, ^116^, ^117^

However, several issues and limitations have to be taken into consideration when developing novel CAR‐T cell‐based therapies in AML. In particular, apart from cost‐related and logistic hurdles, CAR‐T cell treatment requires an extensive knowledge about the technique of CAR and about various practical and technical details. As a result, CAR‐T or CAR‐NK cell approaches can only be developed in highly specialized centers where AML is in focus of preclinical and clinical research. Second, a number of clinically relevant adverse events can occur during CAR‐T or CAR‐NK cell therapy in AML, such as a leukemia cell‐lysis syndrome, a CRS, sustained cytopenia (aplasia), and additional “on‐target but off‐leukemia” toxicities. One major problem is prolonged myelotoxicity especially when the CAR‐T approach will also eliminate normal stem cells.^82^, ^107^, ^108^, ^109^, ^110^, ^111^, ^112^, ^113^, ^114^, ^115^, ^116^, ^117^ This problem may occur with CAR‐T cells directed against diverse stem cell antigens, including CD33. Several strategies have been proposed to overcome this form of toxicity. One is to combine the CAR‐T cell approach with HSCT.^117^ A futuristic idea is to genetically inactivate CD33 in hematopoietic stem cells to enable application of CD33‐directed CAR‐T cell therapy.^118^

Another important issue in CAR‐based AML therapy is that current approaches are not addressing the diversity and the related clonal complexity of AML LSC, but rather focus on only one or two cell surface targets.^107^, ^108^, ^109^, ^110^, ^111^, ^112^, ^113^, ^114^, ^115^, ^116^, ^117^ In this regard, it is important to recognize that AML LSC are heterogeneous cells with varying combinations of cell surface antigens and target structures.^14^, ^15^, ^16^, ^82^ Since all of these subclones can theoretically cause a relapse, it might be of utmost importance to develop poly‐targeted CAR‐T or CAR‐NK cell strategies. A related problem is that AML LSC exhibit multiple forms of LSC resistance.^13^, ^14^, ^15^, ^16^, ^17^, ^18^, ^19^, ^82^ In line with this notion, AML patients relapse quite frequently after CAR‐T or CAR‐NK cell therapy. Therefore, one strategy for the future could be to direct the CAR‐T or CAR‐NK approach against multiple LSC‐related cell surface targets on AML LSC, with the aim to attack most or all AML LSC‐subsets and thus most or all subclones. An important question that remains here is whether such strategy would still spare normal stem cells, even if all the targets that have been selected are largely LSC‐specific. Another open question is when the CAR‐T or CAR‐NK cells should be applied in patients with AML. Based on the toxicity‐profile and the ability of CAR‐T and CAR‐NK cells to eliminate even residual dormant LSC, one strategy may be to apply CAR cells in AML patients at the stage of minimal residual disease. Another strategy would be to apply CAR cells early in AML or even in patients with pre‐AML conditions (myelodysplastic or myeloproliferative neoplasms) with the hope that LSC are more responsive in these patients than in patients with progressed, secondary, or relapsed AML.

## 7 MOBILIZING NK CELLS OR T CELLS AGAINST AML (STEM) CELLS

In a majority of the patients with AML, the production and activity of T and NK cells are substantially impaired compared to T/NK cells in healthy individuals. Based on this notion, a number of therapeutic strategies have been coined with the idea to promote or even restore T cell and/or NK cell production and T/NK cell activity in AML patients and to mobilize these cells specifically against leukemic (stem) cells.^119^, ^120^, ^121^, ^122^, ^123^, ^124^, ^125^ One such strategy is to expand allogeneic NK cell subsets *in vitro* and to infuse these cells in AML patients together with donor stem cells during HSCT.^120^, ^121^, ^122^, ^123^ In fact, alloreactive NK cells are well known to attack leukemic blasts, to improve engraftment, and to promote the graft‐vs‐leukemia effects of the immune system in AML patients undergoing allogeneic HSCT.^122^, ^123^, ^124^, ^125^ A number of different studies using haplo‐identical NK cell infusions have been reported in patients with refractory/relapsed AML. In one of these trials, eight patients with AML or MDS following prior HSCT underwent lympho‐depletion and received donor NK cell infusions and interleukin (IL)‐2.^122^ Although one patient achieved a CR, no benefit concerning OS was reported and the donor NK cells were not detected after infusion in these patients.^122^ All in all, the antileukemic effects of NK cells in patients with AML are well documented, but there is still a debate about the optimal approach to activate these cells against AML LSC. In addition, it remains open how and when NK cell infusions should be applied in these patients to obtain optimal results. An interesting strategy is to induce antibody‐mediated NK cell activity against AML (stem) cells. Indeed, NK cells exhibit antibody‐dependent cellular cytotoxicity, and various antibodies (eg, CD133 or anti‐NKG2A) have been described to mobilize NK cells against AML cells.^126^, ^127^, ^128^, ^129^

As mentioned above, a number of attempts have been made to develop CAR‐NK cells against AML and AML LSC. However, it remains unknown whether CAR‐NK cells can better eliminate AML (stem) cells compared to CAR‐T cells.^130^ Another idea is to apply or to boost both, NK cells and T cells, with the idea to augment antileukemic effects by cooperative NK/T cell effects. Whether such an approach would produce synergistic antileukemic effects remains at present unknown.

In recent years, a number of efforts have been performed to activate (prime) NK cells with stimulating cytokines and to transform these cells into optimized killer cells capable of attacking and eliminating AML LSC. Cytokine‐induced killer cells (CIK) are in general generated by exposing blood lymphocytes to IL‐1, IL‐2 and/or IL‐15. Usually CIK are heterogeneous populations of killer cells exhibiting both T‐cell (CD3) and NK (CD56) cell markers. These CIK have strong antileukemic effects and are able to introduce cytotoxicity against leukemic (stem) cells in an MHC‐restricted and an MHC‐unrestricted manner. A number of preclinical studies have been conducted with CIK in AML and several clinical phase I trials using CIK in refractory/relapsed AML patients have been performed.^131^, ^132^, ^133^, ^134^ In most studies, application of CIK was found to be a feasibility approach with acceptable toxicity. In addition, the efficacy and antileukemic effects of CIK could be demonstrated in some of the patients with relapsed/refractory AML.^131^, ^132^ However, the CIK approach is not efficacious in all patients with AML. Therefore, novel strategies around CIK are currently being explored. One strategy is to combine the CIK approach with a CAR‐T cell strategy.^134^ Whether such combined CIK‐CAR‐T cell therapies are indeed able to kill and eliminate AML LSC remains to be examined in clinical studies.

A more conventional and historical way to boost antileukemic effects of immune cells is to induce NK‐ and T‐cell expansion and activation by infusing IL‐2. In earlier studies, however, no clinically meaningful effects of IL‐2 were observed in patients with AML. This may be due to the fact that immune cell responses against IL‐2 were suppressed by radical oxygen species (ROS) produced by accessory cells or immune cells. To overcome the ROS‐mediated suppression of immune cells, IL‐2 infusions were combined with histamine injections to block production of ROS.^135^, ^136^, ^137^ Such clinical studies with IL‐2 plus histamine were conducted in AML patients in CR as maintenance approach. Although the protocol has practical issues, the approach showed convincing results with an improved OS seen in CR patients treated with IL‐2 + histamine.^136^, ^137^ As a result, this drug combination (IL‐2 plus histamine) was approved as maintenance therapy in AML patients in CR by the European Medicines Agency (EMA) in 2008. More recent data suggest that this maintenance therapy is mostly effective in AML patients with normal karyotype.^138^ However, individual patients with core binding factor mutated AML or *FLT3*‐mutated AML in CR may also benefit from this therapy.^82^ It has also been described that IL‐2 and histamine can mobilize the NK‐ and T‐cell system in these patients.^139^, ^140^ However, it remains unknown whether such maintenance approach is in general efficacious and whether during this therapy minimal residual disease in AML can disappear. Moreover, patients and doctors have to be trained in detail for this therapy to avoid adverse reactions to IL‐2 and/or histamine. In fact, both agents, when injected too quickly, can induce serious adverse events, including hypotension, tachycardia, and shock. Another related issue is that this therapy has to be performed over a certain time‐period. As a result, IL‐2 + histamine is not broadly used as maintenance therapy in AML patients although some efficacy has been demonstrated.

## 8 RESISTANCE OF AML LSC AGAINST IMMUNOTHERAPIES

One of the major problems in the treatment of AML is resistance of immature progenitor cells and stem cells against various therapies.^14^, ^15^, ^16^, ^17^, ^18^, ^82^ In fact, AML LSC are considered to exhibit multiple forms of resistance and several of these resistance mechanisms may play a role in the failure to respond to immunotherapies.^82^ Moreover, AML LSC are heterogeneous populations of cells with varying molecular aberration profiles and multiple, independent (subclone‐specific) resistance mechanisms in individual patients.^15^, ^16^, ^17^ An overview of resistance mechanism relevant to AML LSC is shown in Table 3. In general, resistance‐types in AML LSC can be divided into: (a) intrinsic stem cell resistance common to LSC in all AML subclones, (b) acquired (secondary) resistance mediated by additional (acquired) somatic mutations, loss of tumor suppressor antigens, or loss of target‐antigens, (c) niche‐induced resistance, and (d) immunologic resistance induced by expression of certain checkpoint molecules, like PD‐L1, on AML LSC (Table 3). Intrinsic resistance is commonly related to LSC quiescence and the expression or lack of certain drug‐transporter antigens. This type of resistance often applies to treatment with conventional antileukemic drugs. A promising approach to overcome the intrinsic form of LSC resistance is to use antibodies, antibody‐toxin‐conjugates, or CAR‐T/CAR‐NK cell strategies directed against surface molecules expressed on dormant LSC. However, not all of the quiescent LSC may express these target antigens, and the target‐negative subclones and their LSC survive such immunotherapies. One possibility to overcome this form of “subclone‐related resistance” would be to administer combinations of targeted drugs, combinations of targeted antibodies with a CAR‐approach, or to combine antibody‐based or CAR‐based therapy with conventional drugs or chemotherapy or a stem cell transplantation approach (Table 3).

**TABLE 3:** Resistance of AML LSC against immunotherapies: mechanisms and strategies

Type of resistance and mechanism	Strategies proposed to overcome resistance
Intrinsic stem cell resistance	Antibody‐based therapies; HSCT; CAR
LSC dormancy	Antibody‐mediated targeting of dormant LSC; activating LSC with cytokines to induce cell cycling (cytokine‐priming)—and combination with chemotherapy or TKI^a^; CAR; HSCT
Expression of efflux‐transports like MDR1	MDR1‐targeting drugs; HSCT; TKI^a^; CAR antibody‐based therapies; HSCT; TKI^a^; CAR
Lack of drug transporters (lack of drug uptake)	Antibody‐based therapies; HSCT; TKI^a^; CAR
Niche‐mediated resistance	Niche cell‐targeting drugs, drug combinations, antibody‐based therapies; HSCT; CAR; TKI^a^
Osteoblastic LSC niche	Osteoblast cell‐targeting drugs and/or BET/MYC‐targeting drugs^b^
Vascular LSC niche	Endothelial cell‐targeting (anti‐angiogenic) drugs
LSC‐retention in niche	Mobilizing drugs (plerixafor); HSCT; CAR; TKI^a^
LSC‐hypermobilization (eg, CD26‐mediated)	Mobilization blocker (eg, gliptins or CD26 Ab); antibody‐based therapies; HSCT; CAR; TKI^a^
Acquired resistance of LSC mutations in subclones	Drug combinations; antibody‐based therapies; HSCT; CAR; TKI^a^
Immune checkpoint‐induced	Checkpoint‐targeting antibodies
resistance of LSC	Checkpoint‐targeting CAR cells or BiTE BET/MYC‐targeting drugs^b^; antibody‐based therapies; HSCT; CAR; TKI^a^
Loss of cell surface targets	Mixtures of antibodies; HSCT; CAR directed against 2 or more surface targets; drug combinations + HSCT or CAR
General immunosuppression	Repeated T/NK cell infusion
Blocked immune cells	Bi‐specific antibodies against LSC and immune effector cells; HSCT; CAR; TKI^a^
Loss of CAR‐T cells or CAR‐NK cells	Repeated infusions of CAR cells; cytokine‐induced expansion of CAR cells; drug combinations; HSCT; TKI^a^
Development of blocking antibodies against CARs	Use of single domain scFvs humanize the scFvs; HSCT; TKI^a^

^a^ So far, a few TKIs have been approved for treatment of FLT3‐mutated AML: these drugs are FLT3‐targeting drugs, including midostaurin and gilteritinib. Midostaurin also acts on KIT D816V, which is frequently detected in patients with CBF^+^ AML.

^b^ Several BET/MYC‐targeting drugs reportedly suppress the cytokine‐dependent and oncogene‐mediated PD‐L1 expression in AML LSC as well as osteoblast‐induced resistance.

Abbreviations: AML, acute myeloid leukemia; BiTE, bi‐specific T cell‐engager; CAR, chimeric antigen receptor; HSCT, hematopoietic stem cell transplantation; LSC, leukemic stem cells; MDR1, multidrug resistance gene product 1; NK cells, natural killer cells; scFvs, single chain variable fragments; TKI, tyrosine kinase inhibitor.

The acquired form of resistance of AML LSC may or may not be responsive to various forms of immunotherapy or cell‐based therapies. For example, it may well be that antibody‐based or cell‐based immunotherapies can overcome mutation‐induced (acquired) resistance of AML LSC against tyrosine kinase inhibitors. However, it may also happen that mutation‐induced (acquired) resistance of AML LSC is associated with a “loss” of a certain surface target, such as CD33. Moreover, treatment of AML patients with CD33‐targeted antibodies or CAR‐T cells may well lead to a selection of CD33‐negative subclones and thus resistance (Table 3).

The immunologic type of resistance of AML LSC is often associated with expression of immune checkpoint molecules, such as PD‐L1, CD47, TIM3, or other checkpoint molecules.^75^, ^76^, ^77^, ^78^, ^79^, ^80^, ^81^, ^82^ Multiple factors and mechanisms may be responsible for expression of such checkpoint molecules on AML LSC. For example, PD‐L1 expression on AML LSC can be induced by oncoproteins, certain cytokines (like IFN‐G and TNF‐A) or by a combination of both. In addition, certain drugs, like the hypomethylating agents, can promote PD‐L1 expression.^79^, ^82^ There are also other, therapy‐related, forms of resistance in AML patients receiving CAR cells that have to be considered. For example, a decrease or loss of CAR‐T or CAR‐NK cells and/or the development of auto‐antibodies against these cells may contribute to resistance (Table 3).

Finally, recent data suggest that niche cell‐mediated resistance of LSC against chemotherapy and targeted drugs plays an important role in AML.^82^ Niche cells contributing to LSC resistance are stromal cells, endothelial cells, and osteoblasts. However, so far, it remains unknown whether niche‐mediated resistance of LSC against immunotherapies may also occur and may play a functional role.

Together, multiple forms of LSC resistance may be relevant in AML and several of these resistance mechanisms may act together to induce overt resistance of an AML patient against immunotherapies (Table 3).^82^ As a result, current research is focusing on novel treatment approaches with the goal to overcome resistance of LSC against therapy. One promising approach is to combine various therapies directed against LSC, including antibody‐based therapies, CAR cells, HSCT, and targeted drugs. With regard to niche‐mediated resistance, a number of strategies targeting niche cells (eg, endothelial cells) or targeting LSC‐niche interactions have been proposed. One approach is to mobilize LSC out of the niche using antibodies against CXRC4 or CD44, with the hope to make LSC more sensitive against certain drugs.^33^, ^68^, ^141^ Another concept is to target LSC and niche cells in a combined approach by applying drugs or drug combinations that can suppress or even kill both cell types. However, so far, no curative concept could be established with such approach, although several antibodies and drug combinations have been tested in clinical trials.

One general problem with drug combinations in AML therapy is toxicity, especially myelotoxicity. In fact, when normal stem cells also express the same cell surface targets, long‐term cytopenia may occur. One approach to solve this problem is to select LSC‐specific surface targets that are not expressed (or only expressed in trace amounts) on normal stem cells. Another approach is to combine immunotherapy with stem cell transplanation. Finally, drugs with different mechanisms of actions and different target pathways can be selected, which often reduces (instead of enhancing) toxicity, especially when these drugs produce synergistic antineoplastic effects on AML cells, so that the concentration of the individual drugs can be reduced.

## 9 CONCLUDING REMARKS AND FUTURE PERSPECTIVES

During the past 10 years, our knowledge about LSC, their target expression profiles, and their interactions with the stem cell niche and with certain immune cells, has increased substantially. At the same time, several new forms of cell therapy and other immunotherapies have been developed, and have recently been tested in patients with refractory or relapsed AML in clinical studies. These treatment approaches include new antibody‐based cytotoxic drug‐conjugates, administration of IL‐2 and histamine, application of CAR‐T or CAR‐NK cells, checkpoint‐specific therapies, and bi‐ or tri‐specific antibodies that recruit immune cells and/or cytokines to their target cells (LSC). In many instances, combinations of immunotherapies and intensive therapy are applied, and sometimes, drugs are multitargeted agents with the aim to achieve a broad effect on many or even most LSC subclones in AML. A similar combination effect is obtained when high‐dose chemotherapy and HSCT are combined with donor lymphocyte infusions, which is typically offered to patients with relapsed or refractory AML. However, in many cases, not all LSC can be eradicated, which is due to the multiple forms of LSC resistance. Another major problem is toxicity produced by stem cell‐directed immunotherapies in AML which is often due to the fact that normal stem cells display the same or almost the same targets compared to AML LSC. Therefore, current research is focusing on the development of new approaches to overcome LSC resistance and the toxicity issues with immunotherapy in AML. Whether these approaches will improve outcomes in AML therapy remains at present unknown.

## CONFLICT OF INTEREST

P.V. received a research grant from Celgene, a research grant from Pfizer and honoraria from Celgene and Pfizer. W.R.S. received honoraria from Celgene. The other authors declared no potential conflicts of interest.

## AUTHOR CONTRIBUTIONS

All authors contributed equally to this study by discussing potential immunological targets in AML and AML LSC, by drafting parts of the manuscript. P.V., W.R.S., W.R.: contribution in project design; K.B., I.S., D.S., D.I., H.H., Y.F., G.E.: contribution in stem cell phenotyping by flow cytometry. All authors approved the final version of the manuscript.

## Supporting information


**Appendix** **S1:** Supplementary Information

## Data Availability

Data sharing is not applicable to this article as no new data were created or analyzed in this study.
