# Supplementary material for: Cell‐based and antibody‐mediated immunotherapies directed against leukemic stem cells in acute myeloid leukemia: Perspectives and open issues
Source: Stem Cells Transl Med. 2020 Jul 13;9(11):1331–43. doi: 10.1002/sctm.20-0147 (PMC7581453; doi:10.1002/sctm.20-0147)
Supplement: Supplementary file 1 — Appendix S1: Supplementary Information [file SCT3-9-1331-s001.pdf]

Supplemental Figures

Supplemental Figure 1

Induction of expression of the checkpoint antigen PD-L1 on AML cells

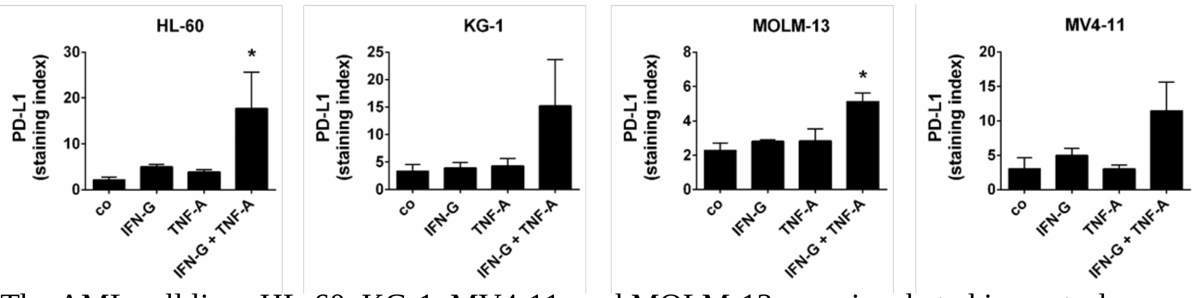

The AML cell lines HL-60, KG-1, MV4-11, and MOLM-13 were incubated in control medium (co) or in medium containing interferon-gamma (IFN-G; 100 U/ml), TNF-alpha (TNF-A; 100 ng/ml) or a combination of IFN-G and TNF-A at 37°C for 24 hours. Then, PD-L1 expression was examined by flow cytometry. Expression levels are provided as staining index (SI) defined by the ratio of median fluorescence intensities (MFI) obtained with PD-L1 antibody and isotype-matched control antibody (SI = MFI PD-L1 antibody : MFI control antibody). Results show the SI values as mean±SD of 3 independent experiments. Asterisk (*): $p<0.05$ compared to control. PD-L1, programmed cell death ligand 1.

Supplemental Figure S2

Expression of CD47, CD80, CD86 and TIM3 on stem and progenitor cells in AML cells (left images) and normal bone marrow (NBM) cells (right images).

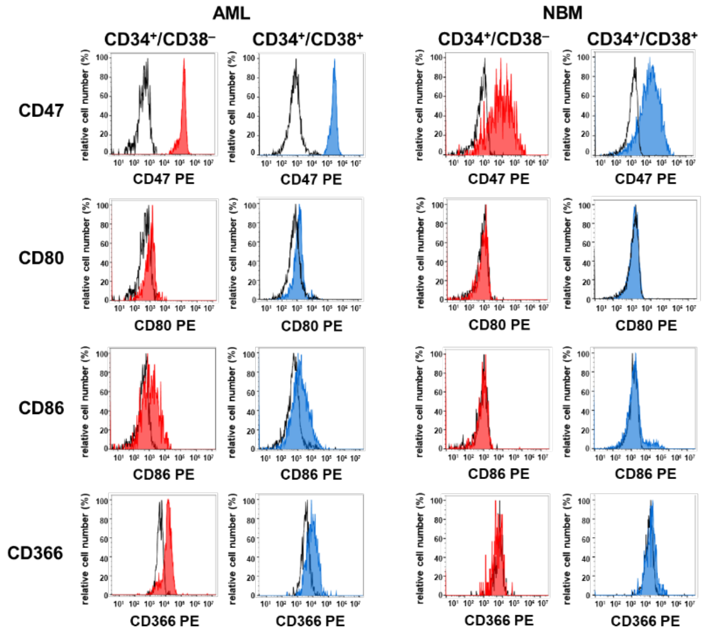

Expression of checkpoint antigens on CD34+/CD38− stem cells (left panels, red histograms) and CD34+/CD38+ progenitors (right panels, blue histograms) was examined by phycoerythrin (PE)-conjugated monoclonal antibodies (mAb – see below) as indicated, and multi-color flow cytometry. All patients gave written informed consent before cells were examined. The study was approved by the ethics committee of the Medical University of Vienna. Antibody-reactivity was assessed on a FACSCanto-II (BD Biosciences) and controlled by isotype-matched control-antibodies (open black histograms). The following PE-labeled mAb were applied: CD47 mAb BH612 (mIgG1), CD80 mAb 2D10 (mIgG1), CD86 mAb IT2.2 (mIgG2b), and the CD366 (anti-TIM3) mAb F38-2E2 (mIgG1).
